# Association between constipation and inguinal hernia: a case-control study in an adult population

**DOI:** 10.1590/0102-67202025000038e1907

**Published:** 2025-10-27

**Authors:** Giorgia MOSTACERO-ROJAS, Jose Antonio CABALLERO-ALVARADO, Katherine LOZANO-PERALTA, Gino VASQUEZ-PAREDES, Joaquin SARMIENTO-FALEN, Victor Eduardo LAU-TORRES, Carlos ZAVALETA-CORVERA

**Affiliations:** 1 Antenor Orrego Private University, Faculty of Medicine – Trujillo, La Libertad, Peru.; 2 Regional Hospital of Trujillo, Department of Surgery – Trujillo, La Libertad, Peru.; 3 Belen Hospital of Trujillo, Department of Neurosurgery – Trujillo, La Libertad, Peru.; 4 Universidad Cientifica del Sur, Faculty of Medicine – Lima, Peru.

**Keywords:** Hernia Inguinal, Constipation, Adult Health, Case-Control Studies, Risk Factors, Hérnia Inguinal, Constipação Intestinal, Saúde Adulta, Estudos de Casos e Controles, Fatores de Risco

## Abstract

**Background:**

Inguinal hernia is the most frequently diagnosed hernia and affects approximately one-third of the male population. Several risk factors have been identified, including advanced age, limited physical activity, smoking, and increased intra-abdominal pressure, among others.

**Aims:**

The aim of the study was to determine whether constipation is a risk factor for inguinal hernia in the adult population.

**Methods:**

A case-control study was conducted at the Department of Surgery of one hospital in the north of Peru, including 121 patients with a confirmed diagnosis of inguinal hernia as cases and 242 patients without such a diagnosis as controls. Inclusion and exclusion criteria were applied, and data were collected through individual interviews using a structured questionnaire that addressed clinical aspects, lifestyles, and the presence of constipation, assessed according to the Rome IV criteria.

**Results:**

The results revealed significant differences between the groups of patients with and without inguinal hernia in terms of age, sex, and anthropometric characteristics. In addition, statistically significant associations were found between the presence of an inguinal hernia and type 2 diabetes, smoking, and constipation. A multivariate analysis showed that age, male sex, body mass index, high blood pressure, and constipation were significant and independent factors associated with the presence of inguinal hernia.

**Conclusions:**

Constipation is a significant risk factor for inguinal hernia in the adult population. These results support the importance of considering constipation as a risk factor in the evaluation and management of patients with inguinal hernia, highlighting the relevance of adequate clinical care in this group of patients.

## ARTICLE HIGHLIGHTS

The global burden of inguinal hernia remains significant, with a higher prevalence in older men and underserved populations.

Risk factors such as collagen disorders, chronic constipation, and prior surgeries contribute to the development of inguinal hernias.

Surgical repair remains the definitive treatment, with growing interest in robotic and laparoscopic techniques for better quality-of-life outcomes.

Despite advances, disparities in diagnosis, management, and postoperative outcomes persist across geographic and socioeconomic settings.

## Summary

**CENTRAL MESSAGE:**

Constipation is a clinically relevant and independent risk factor for inguinal hernia. This association, identified through multivariate analysis in a case–control study of adult patients, underscores the impact of elevated intra-abdominal pressure on abdominal wall integrity. The image provided visually reinforces the strength of this association and the distribution of risk factors.

**PERSPECTIVES:**

The findings from this study highlight constipation as an independent and significant risk factor for inguinal hernia, alongside age, male sex, high body mass index, and hypertension. These results are clinically relevant and may influence future risk stratification and preventive strategies in general surgery and primary care. By addressing modifiable factors such as constipation, healthcare providers could reduce the incidence or delay the onset of inguinal hernias, especially in high-risk populations. This could lead to improvements in patient outcomes, resource use, and surgical burden.

## VISUAL ABSTRACT

**Figure:**
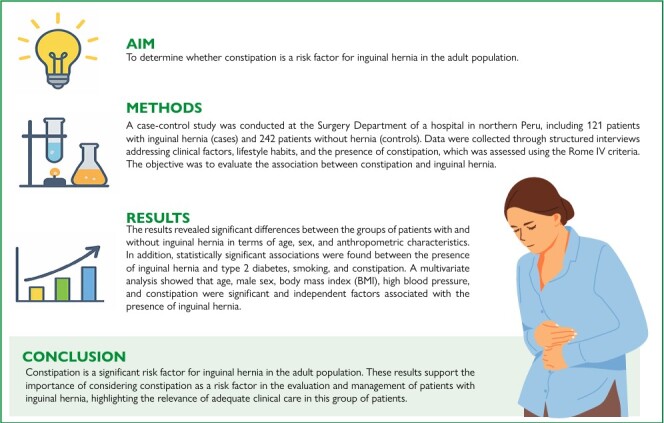

## RESUMO

**Racional:**

A hérnia inguinal é a hérnia mais frequentemente diagnosticada e afeta aproximadamente um terço da população masculina. Diversos fatores de risco foram identificados, incluindo idade avançada, atividade física limitada, tabagismo e aumento da pressão intra-abdominal, entre outros.

**Objetivos:**

Determinar se a constipação é um fator de risco para hérnia inguinal na população adulta.

**Métodos:**

Um estudo de caso-controle foi conduzido no Departamento de Cirurgia de um hospital no norte do Peru, incluindo 121 pacientes com diagnóstico confirmado de hérnia inguinal como casos e 242 pacientes sem tal diagnóstico como controles. Critérios de inclusão e exclusão foram aplicados, e os dados foram coletados por meio de entrevistas individuais usando um questionário estruturado que abordou aspectos clínicos, estilos de vida e presença de constipação, avaliados de acordo com os critérios de Roma IV.

**Resultados:**

Os resultados revelaram diferenças significativas entre os grupos de pacientes com e sem hérnia inguinal em termos de idade, sexo e características antropométricas. Além disso, foram encontradas associações estatisticamente significativas entre a presença de hérnia inguinal e diabetes tipo 2, tabagismo e constipação. Uma análise multivariada mostrou que idade, sexo masculino, índice de massa corporal (IMC), pressão alta e constipação foram fatores significativos e independentes associados à presença de hérnia inguinal.

**Conclusões:**

A constipação é um fator de risco significativo para hérnia inguinal na população adulta. Esses resultados reforçam a importância de considerar a constipação como um fator de risco na avaliação e no tratamento de pacientes com hérnia inguinal, destacando a relevância do cuidado clínico adequado nesse grupo de pacientes.

## INTRODUCTION

Inguinal hernia is the most frequently diagnosed hernia and affects approximately one-third of the male population. It has a bimodal distribution, with a higher incidence in childhood and after age 50^5^. Worldwide, more than 20 million patients undergo inguinal hernia repair annually^9^. In the United States, prevalence varies between 5 and 10%^1^. Pathophysiology involves anatomical and biomechanical factors^3^, such as the protrusion of abdominal contents through weaknesses in the inguinal region, where structures such as the spermatic cord in men and the round ligaments in women converge^20^.

 Several risk factors have been identified, including advanced age, limited physical activity, smoking, and increased intra-abdominal pressure, among others^11,15,18,19^. Recent studies have explored the possible association between chronic constipation and inguinal hernia. It is postulated that prolonged constipation could increase intra-abdominal pressure, progressively weakening the abdominal wall and predisposing it to hernial protrusion^4^. In addition, vigorous defecatory straining may increase tension on the inguinal structures, facilitating protrusion of abdominal contents into the inguinal canal^2^. 

 Research such as that conducted by Kibret et al.^12^ and Idiz and Cakir^10^ has demonstrated significant associations between constipation and the presence of inguinal hernia in cross-sectional and case-control studies. These findings highlight the need for further research into the understanding of this potentially pathogenic relationship. Despite these advances, the current medical literature presents limitations regarding specific studies that investigate this association in a comprehensive manner, thus justifying the performance of new research to clarify the association of constipation as a risk factor for inguinal hernia in the adult population. 

## METHODS

### Study design

 An observational case–control study was conducted from August to December 2023. 

### Population and sample

 Patients seen in the outpatient clinic and inpatient surgery department who were admitted for a surgical procedure. Cases and controls were selected based on the presence of inguinal hernia. A sample size was calculated using the statistical formula for case–control studies, resulting in 121 cases with inguinal hernia and 242 controls without this condition. Inclusion criteria were patients of both sexes, aged 18–65 years, with or without a diagnosis of inguinal hernia confirmed by a surgeon, and exclusion criteria were patients with specific conditions such as cancer, liver cirrhosis, a history of previous abdominal surgery, femoral or bilateral hernias, and recent significant weight loss who attended in the surgery department. 

### Procedures

 Informed consent was obtained from all participants before inclusion in the study. Details, objectives, and procedures were explained (Table 1). Individual interviews were conducted using a structured questionnaire addressing clinical aspects, lifestyles, and the presence of constipation, assessed according to the Rome IV criteria. 

**Table 1:** STrengthening the Reporting of Observational studies in Epidemiology Statement-checklist of items that should be included in reports of observational studies.

		Item No.	Recommendation
Title and abstract		1	(a) Indicate the study’s design with a commonly used term in the title or the abstract
			(b) Provide in the abstract an informative and balanced summary of what was done and what was found
Introduction			
	Background/rationale	2	Explain the scientific background and rationale for the investigation being reported
	Objectives	3	State-specific objectives, including any prespecified hypotheses
Methods			
	Study design	4	Present key elements of the study design early in the paper
	Setting	5	Describe the setting, locations, and relevant dates, including periods of recruitment, exposure, follow-up, and data collection
	Participants	6	(a) Cohort study – Give the eligibility criteria and the sources and methods of selection of participants. Describe methods of follow-up Case-control study – Give the eligibility criteria and the sources and methods of case ascertainment and control selection. Give the rationale for the choice of cases and controls Cross-sectional study – Give the eligibility criteria and the sources and methods of selection of participants
			(b) Cohort study – For matched studies, give the matching criteria and the number of exposed and unexposed Case-control study – For matched studies, give the matching criteria and the number of controls per case
	Variables	7	Clearly define all outcomes, exposures, predictors, potential confounders, and effect modifiers. Give diagnostic criteria, if applicable
	Data sources/measurement	8*	For each variable of interest, give sources of data and details of methods of assessment (measurement). Describe the comparability of assessment methods if there is more than one group
	Bias	9	Describe any efforts to address potential sources of bias
	Study size	10	Explain how the study size was arrived at
	Quantitative variables	11	Explain how quantitative variables were handled in the analyses. If applicable, describe which groupings were chosen and why
	Statistical methods	12	(a) Describe all statistical methods, including those used to control for confounding
			(b) Describe any methods used to examine subgroups and interactions
			(c) Explain how missing data were addressed
			(d) Cohort study – If applicable, explain how loss to follow-up was addressed Case-control study – If applicable, explain how the matching of cases and controls was addressed Cross-sectional study –If applicable, describe analytical methods taking account of sampling strategy
			(e) Describe any sensitivity analyses
Results			
	Participants	13*	(a) Report numbers of individuals at each stage of study – e.g., numbers potentially eligible, examined for eligibility, confirmed eligible, included in the study, completing follow-up, and analyzed
			(b) Give reasons for non-participation at each stage
			(c) Consider the use of a flow diagram
	Descriptive data	14*	(a) Give characteristics of study participants (e.g., demographic, clinical, social) and information on exposures and potential confounders
			(b) Indicate the number of participants with missing data for each variable of interest
			(c) Cohort study – Summarize follow-up time (e.g., average and total amount)
	Outcome data	15*	Cohort study – Report numbers of outcome events or summary measures over time
			Case–control study – Report numbers in each exposure category, or summary measures of exposure
			Cross-sectional study – Report numbers of outcome events or summary measures
	Main results	16	(a) Give unadjusted estimates and, if applicable, confounder-adjusted estimates and their precision (e.g., 95% confidence interval). Make clear which confounders were adjusted for and why they were included
			(b) Report category boundaries when continuous variables were categorized
			(c) If relevant, consider translating estimates of relative risk into absolute risk for a meaningful time period
	Other analyses	17	Report other analyses done – e.g., analyses of subgroups and interactions and sensitivity analyses
Discussion			
	Key results	18	Summarize key results with reference to study objectives
	Limitations	19	Discuss limitations of the study, taking into account sources of potential bias or imprecision. Discuss both the direction and the magnitude of any potential bias
	Interpretation	20	Give a cautious overall interpretation of results, considering objectives, limitations, multiplicity of analyses, results from similar studies, and other relevant evidence
	Generalizability	21	Discuss the generalizability (external validity) of the study results
Other information			
	Funding	22	Give the source of funding and the role of the funders for the present study and, if applicable, for the original study on which the present article is based

* Give information separately for cases and controls in case-control studies and, if applicable, for exposed and unexposed groups in cohort and cross-sectional studies.

### Ethical aspects

 The study was approved by the Antenor Orrego Private University and the Bioethics Committee of the same university (number 1804847335055818752). The ethical standards of Helsinki and CIOMS (Council for International Organizations of Medical Sciences), as well as national regulations, were followed. 

### Data analysis

 SPSS version 28 statistical software (Statistical Package for the Social Sciences) was used for data analysis. Measures of central tendency and dispersion, as well as frequencies and percentages, were calculated. The ꭓ^2^ test was used for categorical variables and the Student’s t-test for quantitative variables. A multivariate analysis was performed using logistic regression to control for confounding factors and to calculate adjusted odds ratios with 95% confidence intervals. 

## RESULTS

 A total of 363 patients were obtained, in a 1:2 ratio. Patients with inguinal hernia (121 cases) and patients without inguinal hernia (242 controls). The mean age was 52.93±9.45 years for cases and 44.78±12.70 years for controls. There was a predominance of males in both groups, 93 (76.86%) for cases and 87 (35.95%) for controls. The mean weight was 73.75±10.55 kg in the case group and 69.49±12.73 kg in controls. The mean body mass index (BMI) was 28.02±3.90 in the case group and 27.38±4.87 in controls. A higher proportion of obese patients was found in controls, 64 (26.45%), compared to cases, 40 (33.06%) (Table 2). 

**Table 2:** Distribution of patients according to general variables and inguinal hernia.

General variables		Inguinal hernia Yes (n=121)	Inguinal hernia No (n=242)	OR (95%CI)	p-value
Age (years)		52.93±9.45	44.78±12.70	NA	0.001
Sex (%)					
	Male	93 (76.86)	87 (35.95)	5.92 [3.60–9.73]	0.001
	Female	28 (23.14)	155 (64.05)		
Weight (kg)		73.75±10.55	69.49±12.73	NA	0.002
Height (m)		1.62±0.07	1.59±0.08	NA	0.001
BMI (kg/m^2^)		28.02±3.90	27.38±4.87	NA	0.211
Categorized BMI (%)					
	Normal weight	29 (23.97)	80 (33.06)	NA	0.168
	Overweight	52 (42.98)	98 (40.50)		
	Obesity	40 (33.06)	64 (26.45)		

BMI: body mass index; NA: not applicable; OR: odds ratio; CI: confidence interval.

 A significant association was found between diabetes mellitus and inguinal hernia (OR 1.97; 95%CI 1.20–3.24, p<0.05). A statistical significance was also found between smoking and inguinal hernia (OR 2.31; 95%CI 1.36–3.94, p<0.05). Constipation was significantly associated with inguinal hernia (OR 5.19; 95%CI 3.23–8.34, p<0.05). However, no statistical significance was found between arterial hypertension, chronic obstructive pulmonary disease, and physical activity in relation to inguinal hernia (Table 3). 

**Table 3:** Distribution of patients according to clinical variables and inguinal hernia presence.

Inguinal hernia				
Clinical variables		Yes (n=121)	No (n=242)	OR (95%CI)	p-value
T2D (%)					
	Yes	39 (32.23)	47 (19.42)	1.97 [1.20–3.24]	0.007
	No	82 (67.77)	195 (80.58)		
HTN (%)					
	Yes	34 (28.10)	54 (22.31)	1.36 [0.83–2.24]	0.225
	No	87 (71.90)	188 (77.69)		
Smoker (%)					
	Yes	34 (28.10)	35 (14.46)	2.31 [1.36–3.94]	0.002
	No	87 (71.90)	207 (85.54)		
COPD (%)					
	Yes	17 (14.05)	34 (14.05)	1.00 [0.53–1.87]	1.000
	No	104 (85.95)	208 (85.95)		
Physical activity (%)					
	Yes	32 (26.45)	73 (30.17)	0.83 [0.51–1.36]	0.461
	No	89 (73.55)	169 (69.83)		
Constipation (%)					
	Yes	71 (58.68)	52 (21.49)	5.19 [3.23–8.34]	0.001
	No	50 (41.32)	190 (78.51)		

T2D: type 2 diabetes; HTN: hypertension; COPD: chronic obstructive pulmonary disease; OR: odds ratio; CI: confidence interval.

 Age (OR 1.05 adjusted 95%CI 1.03–1.08, p<0.05), male sex (OR 8.96 adjusted 95%CI 4.81–16.69, p<0.005), body mass index (OR 1.07 adjusted 95%CI 1.01–1.14, p<0.05), hypertension (OR 2.27 adjusted 95%CI 1.15–4.48, p<0.05) and constipation (OR 8.39 adjusted 95%CI, 4.38–16.05, p<0.005) are significant independent factors associated with the presence of inguinal hernia (Table 4). 

**Table 4:** Multivariate analysis of independent predictors for inguinal hernia.

Predictor	B	Wald	p-value	Adjusted OR (ORa)	95%CI lower	95%CI upper
Age (years)	0.05	15.05	<0.001	1.05	1.03	1.08
Male sex	2.19	47.71	<0.001	8.96	4.81	16.69
BMI (kg/m^2^)	0.07	4.54	0.033	1.07	1.01	1.14
Hypertension	0.82	5.63	0.018	2.27	1.15	4.48
Constipation	2.13	41.27	<0.001	8.39	4.38	16.05
Constant	-6.98					

BMI: body mass index; Ora: adjusted odds ratio; CI: confidence interval.

## DISCUSSION

 The prevalence of inguinal hernias (protrusions of organs or fatty tissue through the inguinal or femoral canal) throughout life ranges between 27 and 43% in men and between 3 and 6% in women^9^. Most are symptomatic, and the only effective solution is surgery. Although a small percentage of patients do not experience symptoms, even in this group, the watch-andwait strategy leads to surgery in approximately 70% of cases within a 5-year period^8^. Worldwide, inguinal hernia repair is one of the most common surgical procedures, performed on more than 20 million people each year^21^. 

 There are several risk factors that influence the development of primary inguinal hernias (PIHs), some of which have been the subject of more extensive research. These risk factors include a wide range of factors, including genetic predisposition (first-degree relatives), gender (prevalence approximately 8–10 times higher in men), age (prevalence at 5 years, mainly of the indirect type, and at 70–80 years, mainly of the direct type), alteration in collagen metabolism (related to a decrease in the proportion of collagen type I/III), and history of prostatectomy (especially if it is radical and performed by open surgery), among others^6,13,14,22^. 

 The present case-control study focused on the evaluation of constipation as a risk factor for the presence of inguinal hernia. Our study found a statistically significant relationship, specifically it was found that patients with inguinal hernia were eight times more likely to have constipation; in this regard, Pivo et al.^17^, in the United States, evaluated the sex-based differences in risk factors for inguinal hernia in adults; of the 494 patients, 202 (40.9%) were women; among the risk factors that stood out in women was constipation; another study conducted in Nigeria by Udo et al.^20^, evaluated the risk factors for inguinal hernia, studied 65 patients and found chronic constipation as a risk factor; another study conducted in India by Agarwal et al.^2^, investigated the risk factors for inguinal hernia in 110 patients, finding that altered bowel habits (constipation) was a relevant risk in this sample, found in almost a third of them. 

 Constipation has been associated with an increase in intraabdominal pressure due to excessive straining during bowel movements. This increased pressure in the abdomen can put force on weaker areas of the abdominal wall, including the inguinal area, which is a region where muscles and tissues may be more susceptible to weakness or defects. When intra-abdominal pressure is repeatedly increased due to chronic constipation and straining during bowel movements, it could contribute to the development or worsening of an inguinal hernia^16^. 

 Other risk factors identified in our research were age, male gender, BMI, type 2 diabetes and smoking; these factors are consistent with other studies; a study conducted in the United States by Cowan et al.^7^, found in a sample of 7,314 patients undergoing inguinal hernia repair during the study period, in a multivariate model, advanced age, male sex, greater vigorous physical activity, alcohol drinking status, and smoking as risk factors; obesity and overweight were associated with a lower incidence of inguinal hernia; Öberg et al.^15^ in Denmark, male sex and older age were also reported as risk factors; smoking increases the risk of recurrence, but it is not known for certain whether it is a risk factor for developing a PIH. 

 It is important to note some limitations of this study. First, the retrospective nature of the case-control design implies the possibility of selection and recall biases in data collection. Furthermore, data were obtained from patients seeking surgical care, which could introduce selection bias and limit the generalizability of the findings to the general population. Furthermore, although significant risk factors were identified, this study was unable to establish definitive causal relationships. Future research with prospective designs and a more representative sample could address some of these limitations and provide a more complete understanding of the risk factors associated with inguinal hernia. 

## CONCLUSIONS

 Constipation is a significant risk factor for inguinal hernia in the adult population. The results of this case-control study suggest that patients with inguinal hernia are more likely to suffer from constipation, in addition to other factors such as older age, male sex, high BMI, and the presence of high blood pressure. These findings underline the importance of considering constipation in the evaluation and management of patients with inguinal hernia. Appropriate clinical care that includes prevention and treatment of constipation could contribute to reducing the risk of inguinal hernia in the population. The evidence generated in this study supports the need for future research that continues to explore this association and develop effective intervention strategies. 

## Data Availability

The information regarding the investigation, methodology, and data analysis of the article is archived under the responsibility of the author
